# Principles of environmental medicine: an educational case study of lead poisoning in Beethoven

**DOI:** 10.3389/fpubh.2025.1578949

**Published:** 2025-07-09

**Authors:** Thomas C. Erren, Florian Glenewinkel, Andreas Pinger, Philip Lewis

**Affiliations:** Institute and Policlinic for Occupational Medicine, Environmental Medicine and Prevention Research, Faculty of Medicine and University Hospital of Cologne, University of Cologne, Cologne, Germany

**Keywords:** Lead poisoning, environment, human biomonitoring, prevention, education

## Abstract

**Background:**

Understanding the nature and consequences of widespread lead poisoning is critical to protecting people from harm.

**Educational case study:**

We review the historical example of hypothesized lead poisoning in the famous composer Beethoven as an educational vector to illustrate principles of environmental medicine. We discuss what would happen if a hypothetical present-day Mr. B with comparable health complaints, clinical symptoms, or test results were to visit a doctor today. Human biomonitoring – with population reference values and health-based guidance values – is discussed as an important diagnostic tool.

**Conclusion:**

This practical outlook on lead poisoning for readers with non-environmental medicine backgrounds contributes to an understanding of principles of environmental medicine.

## Introduction

Lead is one of the biggest environmental health problems in terms of the number of people exposed and the impact on public health all across the globe (1). The World Health Organisation notes that environmental pollution has led to lead poisoning and associated health problems in high, middle, and low-income countries (1). While lead poisoning can occur in any region, it is particularly problematic in the global south (2, 3). To understand the sources and consequences of environmental lead poisoning requires knowledge of environmental medicine. Therefore, educating and training physicians and students with non-environmental medicine backgrounds and increasing awareness and understanding beyond medical specialists is important.

Herein, our aim is to provide information on how environmental medicine as a discipline can help determine whether lead causes or contributes to certain ailments and diseases based on measurements and patient information. To this end, we use the composer Ludwig van Beethoven (1770–1827) and his hypothesized lead poisoning as an educational vector and we discuss what would happen if a hypothetical Mr. B with comparable clinical signs, health complaints, and test results were to visit a doctor today. We choose Beethoven for two reasons. First, the case is topical. In 2024, a CNN report stated: “New analysis of Beethoven’s hair reveals possible cause of mysterious ailments, scientists say” and “Beethoven’s hair reveals new insights into the composer’s health issues” (4, 5). Second, the hair analyses are useful for illustration of environmental medicine principles.

We expect that using the famous figure of Beethoven and a hypothetical modern day Mr. B will contribute to understanding principles of environmental medicine that can benefit individuals and populations today (6).

### Lead

Lead is a toxic metal that is naturally found and widely distributed in the environment. It has been used for thousands of years and in various applications due to its many useful properties (e.g., soft, low melting point, high density, corrosion resistance). Such applications range from writing tablets, water pipes, and ballast in boats to batteries and protection against radiation (7). It has even been added to food and wine as it was used to make a sweetener (lead acetate, “lead sugar”) (8). Drinking water that flows through water distribution systems with lead pipes and lead plumbing can be a significant source of exposure to this environmental toxin today (9). In addition to drinking water and, indeed, occupational sources (10–12), leaded gasoline, canned foods with soldered joints, lead-based paints, and traditional medicines are also relevant sources of environmental lead exposure (7, 13, 14). Lead can be found in dust as paint and pipes deteriorate over time or are subject to renovations, or from mining, coal emissions, and smelting activities (15–17). Lead has even be found in children’s toys (18, 19).

It is no surprise then that lead poisoning (ancient name: saturnism) has occurred repeatedly. The negative effects on health are numerous and can include developmental disorders or permanent damage to various organs and systems (especially the brain and nervous system) in children. Acute effects in adults include vomiting and abdominal pain. Long-term effects include anemia and an increased risk of high blood pressure and kidney damage. In pregnant women, high levels of lead have been linked to miscarriages, stillbirths, premature births, and low birth weight (1). The WHO lists lead as one of the 10 chemicals that are particularly important for public health worldwide (20).

## Methods

Firstly, we non-systematically screened the published literature (via PubMed and Google Scholar) and grey literature (via Google) for information on measurements of lead in Beethoven. We provide a synthesis of the narrative surrounding analyses of Beethoven’s hair samples and ailments that can be used to illustrate several principles of environmental medicine.

Secondly, we introduce human biomonitoring as a diagnostic tool. Comparison of the lead values reported for Beethoven to population reference values and health-based guidance values for environmental exposures allows answering a series of questions that clarify how human biomonitoring for Beethoven and a present-day Mr. B corresponds with today’s principles of environmental medicine.

## Results

### Information on measurements of lead in Beethoven

Evidence supporting the hypothesized lead poisoning of Beethoven was announced in 2000 and was based on hair analyses (21, 22). A high concentration of lead (mean ~60 ppm) was identified in the so-called Hiller lock (the composer Ferdinand Hiller cut locks of hair from Beethoven in the days around his death) (22, 23). This four-year project to investigate the cause of Beethoven’s mysterious chronic illness (including digestive problems, chronic abdominal pain, hearing loss, irritability, and depression) and death concluded that “lead poisoning” was the likely answer (21, 22). In 2023 though, a genomic study that involved eight locks attributed to Beethoven revealed that the Hiller lock was from a woman (24).

Five of the eight locks tested in 2023 were determined as originating from Beethoven (24). Among these, the Bermann Lock and the Halm-Thayer Lock were analysed by Rifai et al. (25) for lead, arsenic, and mercury. The measured hair lead concentration and the predicted blood concentration corresponding to the Halm-Thayer Lock that they reported are presented in Table 1. To estimate Beethoven’s blood lead concentration based on the Halm-Thayer Lock value, Rifai et al. used a conversion formula based on hair and blood concentrations from historical data at the Mayo Clinic, Rochester, MN. Their result for a predicted blood lead concentration – namely, 69–71 μg/dL –can suffice for illustrative purposes.

**Table 1:** Lead assessed in Beethoven’s hair and blood, population reference values and health-based guidance values, and WHO guideline for the clinical management of exposure to lead (chelation therapy).

Beethoven	
Hair (Halm-Thayer lock, measured) (25)	369–380 μg/g
Blood (predicted from hair) (25)	69–71 μg/dL
Population reference values	
2022, USA CDC 97.5th percentile (52)	3.5 μg/dL
2019, Germany Human Biomonitoring Commission 95^th^ percentile – Adult Women (26)	3 μg/dL
2019, Germany Human Biomonitoring Commission 95^th^ percentile – Adult Men (26)	4 μg/dL
2003, Germany Human Biomonitoring Commission 95^th^ percentile – Adult Women (26)	7 μg/dL
2003, Germany Human Biomonitoring Commission 95^th^ percentile – Adult Men (26)	9 μg/dL
Health-based guidance values	
1996, Germany Human Biomonitoring Commission HBM-I (53)	15 μg/dL*
1996, Germany Human Biomonitoring Commission HBM-II (53)	25 μg/dL*
WHO guideline for clinical management of exposure to lead 2021, Chelation therapy for Adults (28)	>70 μg/dL**
2025, USA CDC guidance - consult specialist and consider chelation therapy (54)	>45 μg/dL**

CDC, Centers for Disease Control.

^*^Designated for “other persons” as different from children ≤12 years and females of reproductive age.

^**^Chelation therapy may be recommended at lower values if the patient is symptomatic or for children.

### Introduction of human biomonitoring as a diagnostic tool

Human biomonitoring involves the examination of human biological samples for toxic substances. Interpretation of blood lead concentration values (i.e., as would be determined today from a blood sample sent to an accredited laboratory) requires comparison with: (a) population reference values and (b) health-based guidance values from toxicology and epidemiology.

Regarding (a), population reference (or background) values can provide statistical orientation, i.e., to allow assessment of exposure relative to other members of the population (but not risk). For instance, the Centers for Disease Control and Prevention–National Institute for Occupational Safety and Health in the USA uses a blood lead reference value of 3.5 μg/dL (based on the 97.5^th^ percentile of the blood lead distribution) above which individuals are identified as having higher levels of lead in their blood compared to most other members of this reference population (26, 27). In Germany, the 95^th^ percentiles of the blood lead distributions for women and men in 2003 were estimated at 7 and 9 μg/dL, respectively (26). Since 2019, these values are in need of updating (still pending) but the German Human Biomonitoring Commission puts forward preliminary values of 3 and 4 μg/dL for women and men as estimates of the 95^th^ percentile, respectively (26). It should be noted that an individual’s relative exposure levels compared to background levels are only inferred. Exposure levels per se could be higher if some of the lead is not absorbed (i.e., exposure does not equate to dose). Nonetheless, comparing to population reference values provides useful statistical orientation.

Regarding (b), for assessment of risk of health effects, health-based guidance values from toxicology/epidemiology, with cultural and political considerations (i.e., “when and how should we act?”), are necessary. In Germany, the Human Biomonitoring Commission provides their HBM-I and HBM-II values (HBM = Human Biomonitoring) as health-based guidance values that can provide medical orientation regarding toxic substances. Health-based guidance values are determined from dose–response relationships and applying safety factors (e.g., dividing by a factor of 10) to mitigate against any uncertainties (e.g., poor quality of evidence regarding dose–response relationship, or translating from rodent studies to humans, or translating from adults to small children or pregnant women). For lead, they are currently suspended and awaiting an update, with the population reference value currently recommended as the benchmark beyond which active exposure reduction should be initiated (27). Medical intervention is often based on internationally respected recommendations such as the WHO guideline for clinical management of exposure to lead (28).

All values described above are presented in Table 1 for comparison. The evolution of population reference and HBM values for lead is useful for illustration of environmental principles and will be covered in the discussion section. See Box 1 for further description of population reference values and for details on the German Human Biomonitoring Commission’s HBM-I and HBM-II values.

BOX 1 **Values of the German Commission for Human Biomonitoring for the assessment of substances in the human body**

**Population reference values** are determined using a series of measured values from a defined sample that is considered representative of a population group. The comparison of a measured value with population reference values (e.g., the median value or the 95^th^ percentile value) means a comparison with the background exposure to lead – or the normal distribution of lead. They do not provide any information about adverse health effects on humans; they merely allow an assessment of exposure.

**HBM-I and HBM-II values** are toxicologically and epidemiologically based, i.e., they are derived from studies with information on the relationship between the concentration of a substance or its metabolites in human body fluids and the occurrence of adverse effects in humans. Such values enable a risk assessment.

- **HBM-I values**: concentration of a compound in human biological material below which no adverse health effects are to be expected. If the HBM-I value is not exceeded, there is no need for action (29).
- **HBM-II values**: concentration in human biological material which, if exceeded, can lead to adverse health effects considered relevant for exposed individuals (30). If the HBM-II value is exceeded, there is need for action.
- Human biomonitoring values between **HBM-I and HBM-II values** mean that adverse health effects cannot be ruled out with certainty: monitoring of the values (analysis, time course), the search for specific sources of exposure and a reduction of exposure with reasonable effort is indicated.

## Discussion

Based on the synthesis of published lead measurements regarding Beethoven and on information about human biomonitoring, we now elaborate on the role and importance of environmental medicine by discussing: (i) What could Beethoven’s measurement results mean? (ii) How would we “treat” the case of a present-day Mr. B? (iii) Are there unexpected sources of environmental lead exposures? (iv) How do population reference values and health-based guidance values evolve? (v) What principles of environmental medicine are identified in our article? (vi) What do we conclude for readers without a background in environmental medicine?

### What could Beethoven’s measurement results mean?

Using the information from Table 1 to inform Beethoven’s risk orientation requires historical context. Indeed, the environmental conditions in the 1700s and 1800s come into play when evaluating the hypothesized health risks of lead for Beethoven. For example, lead contamination in food and drink was quite common for – at least parts of – the population at that time (7). Further candidates for lead exposures in Beethoven’s day include lead-containing medical treatments (7, 25). It is therefore very likely that the reference values for populations in the 1700s and 1800s would be quite different from today. The locks that were mistaken for Beethoven’s actually indicate that there were others with similar hair lead concentrations in his time (21, 22). In other words, they could be indicative of high background levels in the population at the time (21). However, lacking information on frequency of disease and complaint combinations such as Beethoven’s for other people in his time, we cannot rule out that his disease history was due to causes other than the hypothesized lead poisoning (8, 31, 32).

### How would we “treat” the case of a present-day Mr. B?

Suppose a Mr. B were to see a doctor today, presenting with complaints and illnesses that could be consistent with lead poisoning. Careful questions (including about Mr. B’s medical history, occupation, environment, and living conditions) might bring the doctor to consider lead poisoning as one differential diagnosis. In the age of Dr. Google and ChatGPT, Mr. B might even request a blood test for lead directly (33–35). If other explanations for the patient’s symptoms and complaints are ruled out or deemed less likely (or sometimes, if a patient is fixated on lead as the cause of their illness), the physician may have Mr. B’s blood analysed at an accredited laboratory. Blood analysis is the gold standard for measuring lead in the human body. Lead levels might also be assessed non-invasively, such as by testing hair, but results from hair are limited in interpretability (different accumulation patterns in hair compared to in blood, possible external contaminations of hair, no widely accepted standard conversion factor from hair to blood).

In the following, we treat Beethoven’s estimated blood lead values as if they were results measured for Mr. B today. These blood lead values can be compared with population reference and health-based guidance values (Table 1). This empirical basis enables two steps of assessing the body burden of toxic substances in humans. To assess Mr. B’s blood lead concentration with respect to exposure, the physician can compare the measurement results to population reference values for the lead concentration in a specified population. This comparison provides a *statistical orientation* – in the case of Mr. B, his blood lead concentrations would be ≈8 times higher than those of 95% of the adult men in Germany’s population in 2003 or ≈17 times higher than in 2019 (Table 1). In short, Mr. B’s lead *exposure* is significantly higher than that of these reference populations. To assess Mr. B’s blood lead concentration with regard to health effects, the physician can compare Mr. B’s blood lead concentration to, for instance, the German Human Biomonitoring Commission’s HBM-I and HBM-II values. This provides a *medical orientation* – in the case of Mr. B, his blood lead concentration would be 2.8 times the HBM-value II (Table 1). In short, Mr. B’s *risk* of adverse health outcomes from lead is very high. Empirically, exposure to lead at this level may contribute to or cause health problems.

In practice, the elevated lead concentration in Mr. B’s blood means that measures are required to identify the responsible lead source(s), to prevent such exposure, and for possible therapy. Possible sources of today’s exposures include drinking water from lead pipes, lead paints, and lead in dust, but there may also be unusual routes of exposure, as shown in the following case series (36–38).

### Are there unexpected sources of environmental lead exposure today?

Yes. In 2007, 29 patients were admitted to several hospitals in the greater Leipzig area of Germany, which has a population of around 650,000 people (37, 38). All patients presented with the classic signs and symptoms of acute lead poisoning, including abdominal cramps, nausea, neurologic symptoms, anemia of varying severity, fatigue, a “Burton’s line,” and basophilic stippling. The diagnosis was made quickly and the chelation therapy was effective – blood lead levels on admission in patients treated in this way ranged from 17.5 μg/dL to 457 μg/dL (briefly, chelation therapy involves administering a “chelating agent” intravenously that binds to the heavy metal, which helps the body to eliminate the heavy metal in urine). Remarkably, after 8 weeks, adulterated marijuana was found to be the cause. An extensive criminal investigation led to the hypothesis that the lead, with its high specific gravity (“weight”), was added to the marijuana to maximize the dealer’s profits. An anonymous screening program conducted by the local health office led to the following results: of 597 marijuana consumers, 27.3% had blood lead levels above the HBM-II value, 12.2% had levels requiring monitoring (above HBM-I), and 60.5% had levels below the HBM-I threshold (38).

Unexpected exposures can also result from the use of certain Ayurvedic medicine and various cosmetic, household, hobby, and leisure products that have made their way by (tourism, immigration, or importation that escapes regulation) into regions where substances are more strictly regulated (39–44).

### How do population reference values and health-based guidance values evolve?

Population reference values for lead as purely statistically defined values are determined using, for example, a suitable reference population and regular environmental surveys (e.g., performed by the German Human Biomonitoring Commission for Germany). In contrast, the derivation of health-based guidance values, such as the HBM-I and HBM-II values (also from the German Human Biomonitoring Commission), requires a rich base of toxicological and epidemiological studies or toxicokinetic extrapolation. Such studies allow to determine concentrations of substances or their metabolites that correspond to tolerable intake doses, which may change as new studies and findings become available. Against this challenging evidence background, the HBM-I and HBM-II values in Germany are available only for a few substances such as cadmium and pentachlorophenol (PCP) (27). At the international level, there is a freely accessible repository for extensive population reference and health-based guidance values worldwide for many chemicals, which also includes the German HBM-I and HBM-II values (45). This curated database from the International Human Biomonitoring Working Group (i-HBM) that is affiliated with the International Society of Exposure Science has been created to simplify the process of screening various sources for appropriate human biomonitoring values (45). Lead – identified as important in their publication – remains to be included on their dashboard (at the time of writing). This illustrates (as does Table 1) that identifying population reference values and health-based guidance values for lead is not always straightforward.

Population reference values will change over time. Reference values for the population in Beethoven’s time would certainly be different from today. Increases in knowledge of adverse effects of lead alongside policy interventions such as bans on lead-based paint and leaded petrol serve to reduce exposures. The application of new scientific information to health-based guidance values can also be exemplified by lead. While the German Human Biomonitoring Commission provided important guidance in the above Leipzig poisoning, since 2010 it has suspended the use of HBM-I and HBM-II values for lead and recommended that the population reference value be used as a concentration benchmark for measures to prevent avoidable exposures (46). This is due to the critical effects of lead on the developing organism even at low concentrations and the classification of inorganic lead and its compounds as Group 2A (probably carcinogenic to humans) by the International Agency for Research on Cancer (IARC) (46). Such a policy also exists in other countries (47).

### What principles of environmental medicine are identified in our article?

The above content illustrates several principles of environmental medicine in Germany and elsewhere: (a) Prevention – illustrated by identifying and eliminating potential sources of exposure and very different exposure situations in Beethoven’s time compared to today. (b) Precautionary principle – illustrated by changes in population reference and HBM values over time and by the modern-day physician who may have a blood lead analysis performed at the patient’s insistence. (c) Hazard identification – illustrated by questions about occupation, environment, and living situation and by the historical context in the case of Beethoven. (d) Exposure assessment – illustrated by the use of population reference values and the Leipzig case series. (e) Risk assessment – illustrated by the use of health-based guidance values and the Leipzig case series. (f) Multidisciplinary approach – illustrated by toxicology and epidemiology in determining population reference and health-based guidance values, clinical environmental medicine to assess individual cases, and public health or preventive environmental medicine to apply knowledge from other health sciences to mitigate environmental contamination and lead poisoning. Of course, this list is not exhaustive.

### What do we conclude for readers without a background in environmental medicine?

Given the ubiquitous sources of exposures to lead, every physician should know principles of environmental medicine as a basis for diagnosis, mitigation, therapy, and prevention (48).

Clearly, these should be combined with common sense. This is well summarized by the English saying “when you hear hooves, think horses and not zebras.” With Beethoven’s case history, his estimated lead values and the historical context, we may consider lead poisoning as the “horse” for some of his ailments. If a modern-day Mr. B were to present with lower blood lead values or fewer ailments, lead poisoning may well be the proverbial zebra and other more likely differential diagnoses should be explored first. Another saying holds that “a patient who has fleas may also have lice,” implying that multiple or comorbid symptoms (such as Beethoven’s hearing loss and abdominal pain (21, 22, 25, 32, 49–51)) are not necessarily caused by one exposure alone.

Overall, this article – with Beethoven as an educational vector – conveys principles of environmental medicine that can benefit individuals and populations in many places.

## References

[1] WHO. Lead Poisoning (2024). Available online at:https://web.archive.org/web/20250206131828/https://www.who.int/health-topics/lead-poisoning#tab=tab_1.

[2] Environmental Protection Agency. United Nations Environment Programme Africa Project (2024). Available online at:https://www.epa.gov/international-cooperation/epa-international-cooperation-lead-pollution#:~:text=United%20Nations%20Environment%20Programme%20Africa,well%20as%20live%20French%20translation.

[3] Ericson B Landrigan P Taylor MP Frostad J Caravanos J Keith J. The global burden of lead toxicity attributable to informal used lead-acid battery sites. Ann Glob Health. (2016) 82:686–99. doi: 10.1016/j.aogh.2016.10.015, PMID: 28283119

[4] Strickland A CNN. Beethoven’s hair reveals new insights into the composer’s health issues (2024). Available online at:https://web.archive.org/web/20250206131926/https://edition.cnn.com/2024/05/11/world/beethoven-science-newsletter-wt-scn/.

[5] Strickland A CNN. New analysis of Beethoven’s hair reveals possible cause of mysterious ailments, scientists say (2024). Available online at:https://web.archive.org/web/20250206131954/https://edition.cnn.com/2024/05/09/world/beethoven-lead-poisoning-scn/index.html.

[6] Rose G. Sick individuals and sick populations. Int J Epidemiol. (1985) 14:32–8. doi: 10.1093/ije/14.1.32, PMID: 3872850

[7] Jonasson ME Afshari R. Historical documentation of Lead toxicity prior to the 20th century in English literature. Hum Exp Toxicol. (2018) 37:775–88. doi: 10.1177/0960327117737146, PMID: 29076389

[8] Eisinger J. Lead and wine. Eberhard Gockel and the Colica Pictonum. Med Hist. (1982) 26:279–302. doi: 10.1017/S0025727300041508, PMID: 6750289 PMC1139187

[9] Hanna-Attisha M LaChance J Sadler RC Champney Schnepp A. Elevated blood lead levels in children associated with the Flint drinking water crisis: a spatial analysis of risk and public health response. Am J Public Health. (2016) 106:283–90. doi: 10.2105/AJPH.2015.303003, PMID: 26691115 PMC4985856

[10] Dounias G Rachiotis G Hadjichristodoulou C. Acute Lead intoxication in a female battery worker: diagnosis and management. J Occup Med Toxicol. (2010) 5:19. doi: 10.1186/1745-6673-5-19, PMID: 20609219 PMC2914075

[11] Mohammadi S Mehrparvar A Aghilinejad M. Appendectomy due to Lead poisoning: a case-report. J Occup Med Toxicol. (2008) 3:23. doi: 10.1186/1745-6673-3-23, PMID: 18928540 PMC2575197

[12] Hart SP McIver B Frier BM Agius RM. Abdominal pain and vomiting in a paint stripper. Postgrad Med J. (1996) 72:253–5. doi: 10.1136/pgmj.72.846.253, PMID: 8733544 PMC2398416

[13] Budnik LT Baur X Harth V Hahn A. Alternative drugs go global: possible Lead and/ or mercury intoxication from imported natural health products and a need for scientifically evaluated poisoning monitoring from environmental exposures. J Occup Med Toxicol. (2016) 11:49. doi: 10.1186/s12995-016-0139-0, PMID: 27833648 PMC5101689

[14] Breeher L Gerr F Fuortes L. A case report of adult Lead toxicity following use of Ayurvedic herbal medication. J Occup Med Toxicol. (2013) 8:26. doi: 10.1186/1745-6673-8-26, PMID: 24083830 PMC3850721

[15] Ikegami A Ohtsu M Sahito A Khan AA Fatmi Z Nakagi Y. Contribution of house dust contamination towards lead exposure among children in Karachi, Pakistan. Rev Environ Health. (2020) 35:271–5. doi: 10.1515/reveh-2020-0020, PMID: 32651990

[16] Hu X Sun Y Ding Z Zhang Y Wu J Lian H. Lead contamination and transfer in urban environmental compartments analyzed by Lead levels and isotopic compositions. Environ Pollut. (2014) 187:42–8. doi: 10.1016/j.envpol.2013.12.025, PMID: 24440437

[17] Ceballos DM Herrick RF Dong Z Kalweit A Miller M Quinn J. Factors affecting Lead dust in construction workers' homes in the greater Boston area. Environ Res. (2021) 195:110510. doi: 10.1016/j.envres.2020.110510, PMID: 33245888

[18] Mateus-Garcia A Ramos-Bonilla JP. Presence of lead in paint of toys sold in stores of the formal market of Bogota, Colombia. Environ Res. (2014) 128:92–7. doi: 10.1016/j.envres.2013.11.005 24359710

[19] Shen Z Hou D Zhang P Wang Y Zhang Y Shi P. Lead-based paint in children's toys sold on China's major online shopping platforms. Environ Pollut. (2018) 241:311–8. doi: 10.1016/j.envpol.2018.05.078, PMID: 29843013

[20] WHO. Chemical Safety and Health. Available online at:https://web.archive.org/web/20240729162949/https://www.who.int/teams/environment-climate-change-and-health/chemical-safety-and-health/health-impacts/chemicals/.

[21] Reiter C. The causes of Beethovens death and his locks of hair: a forensic-toxicological investigation. Beethoven J. (2007) 22:1–5.

[22] APS. The Advanced Photon Source: Aps Analysis of Beethoven Hair Sample Yields Clues to Composer's Life and Death (2000). Available online at:https://web.archive.org/web/20250206132031/https://www.aps.anl.gov/APS-News/2017/aps-analysis-of-beethoven-hair-sample-yields-clues-to-composers-life-and-death.

[23] Beethoven MR. *Beethoven's* hair: An extraordinary historical odyssey and a scientific mystery solved. New York, USA: Random House Inc (2001).

[24] Begg TJA Schmidt A Kocher A Larmuseau MHD Runfeldt G Maier PA. Genomic analyses of hair from Ludwig van Beethoven. Curr Biol. (2023) 33:1431–1447.e22. doi: 10.1016/j.cub.2023.02.041, PMID: 36958333

[25] Rifai N Meredith W Brown K Erdahl SA Jannetto PJ. High Lead levels in 2 independent and authenticated locks of Beethoven's hair. Clin Chem. (2024) 70:878–9. doi: 10.1093/clinchem/hvae054, PMID: 38709835

[26] UBA. Aktualisierung Der Referenzwerte Für Blei Im Blut Von Erwachsenen - Stellungnahme Der Kommission Human Biomonitoring Des Umweltbundesamtes. Bundesgesundheitsbl. (2019) 62:1280–4. doi: 10.1007/s00103-019-03002-z 31535275

[27] UBA. Beurteilungswerte Der Hbm-Kommission (2024). Available online at:https://web.archive.org/web/20250206132747/https://www.umweltbundesamt.de/themen/gesundheit/kommissionen-arbeitsgruppen/kommission-human-biomonitoring/beurteilungswerte-der-hbm-kommission.

[28] WHO. Guideline for clinical management of exposure to lead: executive summary. Geneva: World Health Organisation (2021). 34787987

[29] Holzer J Lilienthal H Schumann M. Human biomonitoring (Hbm)-I values for perfluorooctanoic acid (Pfoa) and perfluorooctane sulfonic acid (Pfos) - description, derivation and discussion. Regul Toxicol Pharmacol. (2021) 121:104862. doi: 10.1016/j.yrtph.2021.104862, PMID: 33444659

[30] Schumann M Lilienthal H Holzer J. Human biomonitoring (Hbm)-ii values for perfluorooctanoic acid (Pfoa) and perfluorooctane sulfonic acid (Pfos) - description, derivation and discussion. Regul Toxicol Pharmacol. (2021) 121:104868. doi: 10.1016/j.yrtph.2021.104868, PMID: 33484797

[31] Montes-Santiago J. The Lead-poisoned genius: saturnism in famous artists across five centuries. Prog Brain Res. (2013) 203:223–40. doi: 10.1016/B978-0-444-62730-8.00009-8 24041283

[32] Shearer PD. The deafness of Beethoven: an audiologic and medical overview. Am J Otol. (1990) 11:370–4. 2240185

[33] Van Bulck L Moons P. What if your patient switches from Dr. Google to Dr. Chatgpt? A vignette-based survey of the trustworthiness, value and danger of Chatgpt-generated responses to health questions. Eur J Cardiovasc Nurs. (2023) 23:95–8. doi: 10.1093/eurjcn/zvad038, PMID: 37094282

[34] Erren TC Lewis P Shaw DM. Brave (in a) New World: an ethical perspective on Chatbots for medical advice. Front Public Health. (2023) 11:1254334. doi: 10.3389/fpubh.2023.1254334, PMID: 37663854 PMC10470018

[35] Welsby P Cheung BMY. Chatgpt. Postgrad Med J. (2023) 99:1047–8. doi: 10.1093/postmj/qgad056, PMID: 37462242

[36] Vandenbroucke JP. In defense of case reports and case series. Ann Intern Med. (2001) 134:330–4. doi: 10.7326/0003-4819-134-4-200102200-00017, PMID: 11182844

[37] Busse F Omidi L Timper K Leichtle A Windgassen M Kluge E. Lead poisoning due to adulterated marijuana. N Engl J Med. (2008) 358:1641–2. doi: 10.1056/NEJMc0707784, PMID: 18403778

[38] Busse FP Fiedler GM Leichtle A Hentschel H Stumvoll M. Lead poisoning due to adulterated marijuana in Leipzig. Dtsch Arztebl Int. (2008) 105:757–62. doi: 10.3238/arztebl.2008.0757, PMID: 19623274 PMC2696942

[39] Gorospe EC Gerstenberger SL. Atypical sources of childhood Lead poisoning in the United States: a systematic review from 1966–2006. Clin Toxicol. (2008) 46:728–37. doi: 10.1080/15563650701481862, PMID: 18608287

[40] Prakash S Hernandez GT Dujaili I Bhalla V. Lead poisoning from an Ayurvedic herbal medicine in a patient with chronic kidney disease. Nat Rev Nephrol. (2009) 5:297–300. doi: 10.1038/nrneph.2009.41, PMID: 19384331

[41] Hore P Sedlar S Ehrlich J. Lead poisoning in a mother and her four children using a traditional eye cosmetic - new York City, 2012-2023. MMWR Morb Mortal Wkly Rep. (2024) 73:667–71. doi: 10.15585/mmwr.mm7330a2, PMID: 39088375 PMC11305410

[42] Porterfield K Hore P Whittaker SG Fellows KM Mohllajee A Azimi-Gaylon S. A snapshot of Lead in consumer products across four us jurisdictions. Environ Health Perspect. (2024) 132:75002. doi: 10.1289/ehp14336, PMID: 39012763 PMC11251510

[43] Ma H Wu L-m Zou Y Li X-a. Non-occupational Lead poisoning associated with traditional Chinese medicine: a case report. Front Public Health. (2022) 10:10. doi: 10.3389/fpubh.2022.938186, PMID: 36176514 PMC9513390

[44] Duan Y Yan L Gao Z Gou Y. Lead poisoning in a 6-month-old infant: a case report. Front Public Health. (2023) 11:1132199. doi: 10.3389/fpubh.2023.1132199, PMID: 37213598 PMC10194651

[45] Nakayama SF St-Amand A Pollock T Apel P Bamai YA Barr DB. Interpreting biomonitoring data: introducing the international human biomonitoring (I-Hbm) working group's health-based guidance value (Hb2gv) dashboard. Int J Hyg Environ Health. (2023) 247:114046. doi: 10.1016/j.ijheh.2022.114046, PMID: 36356350 PMC10103580

[46] Wilhelm M Heinzow B Angerer J Schulz C. Reassessment of critical Lead effects by the German human biomonitoring commission results in suspension of the human biomonitoring values (Hbm I and Hbm ii) for Lead in blood of children and adults. Int J Hyg Environ Health. (2010) 213:265–9. doi: 10.1016/j.ijheh.2010.04.002, PMID: 20493765

[47] Ruckart PZ Jones RL Courtney JG LeBlanc TT Jackson W Karwowski MP. Update of the blood lead reference value - United States, 2021. MMWR Morb Mortal Wkly Rep. (2021) 70:1509–12. doi: 10.15585/mmwr.mm7043a4, PMID: 34710078 PMC8553025

[48] Moon J Yoo H. Misdiagnosis in occupational and environmental medicine: a scoping review. J Occup Med Toxicol. (2021) 16:33. doi: 10.1186/s12995-021-00325-z, PMID: 34429147 PMC8383455

[49] Stevens MH Jacobsen T Crofts AK. Lead and the deafness of Ludwig van Beethoven. Laryngoscope. (2013) 123:2854–8. doi: 10.1002/lary.24120, PMID: 23686526

[50] Brotto D Fellin R Sorrentino F Gheller F Trevisi P Bovo R. A modern case sheds light on a classical enigma: Beethoven's deafness. Laryngoscope. (2021) 131:179–85. doi: 10.1002/lary.28464, PMID: 31904878

[51] Rzymski P Zarebska-Michaluk D Flisiak R. Beethoven's deafness more likely linked to viral infection than Lead poisoning. J Infect. (2024) 88:210. doi: 10.1016/j.jinf.2023.12.011, PMID: 38141786

[52] Council of State and Territorial Epidemiologists Position Statement. 22-eh-01: Public health reporting and national notification for lead in blood (2022). Available online at:https://web.archive.org/web/20250206132057/https://cdn.ymaws.com/www.cste.org/resource/resmgr/ps/ps2022/22-EH-01_Lead_in_Blood.pdf.

[53] UBA. Human Biomonitoring Commission (Kommission Human-Biomonitoring Des Umweltbundesamtes); Stoffmonographie Blei-Referenz- Und Human-Biomonitoring-Werte (Hbm). Bekanntmachung Des Instituts Für Wasser-, Boden- Und Lufthygiene Des Umweltbundesamtes. Bundesgesundhbl. (1996) 39:236–41.

[54] CDC. Recommended actions based on blood lead level (2025). Available online at: https://web.archive.org/web/20250620140125/https://archive.cdc.gov/www_atsdr_cdc_gov/csem/leadtoxicity/patient_treatment.html.

